# Pregnancy, prescription medicines and the potential risk of herb-drug interactions: a cross-sectional survey

**DOI:** 10.1186/s12906-017-2052-1

**Published:** 2017-12-19

**Authors:** James S. McLay, Naila Izzati, Abdul R. Pallivalapila, Ashalatha Shetty, Binita Pande, Craig Rore, Moza Al Hail, Derek Stewart

**Affiliations:** 1 The Division of Applied Health Sciences, The University of Aberdeen, Aberdeen, UK; 2 Pharmacy Department, Women’s Hospital-HMC, Doha, Qatar; 3 The Department of Obstetrics and Gynaecology, The University of Aberdeen, Aberdeen, UK; 4 The Department of Obstetrics and Gynaecology, Ninewells Hospital and Medical School, NHS Tayside, Dundee, UK; 5 The Department of Pharmacy, Aberdeen Royal Infirmary, Aberdeen, UK; 6 School of Pharmacy and Life Sciences. Robert Gordon University, Aberdeen, UK

**Keywords:** Herbal, Botanical, Pregnancy, Herb-drug interaction, Antenatal, Postnatal, Female, Surveys and questionnaires

## Abstract

**Background:**

Pregnant women are routinely prescribed medicines while self-medicating with herbal natural products to treat predominantly pregnancy related conditions. The aim of this study was to assess the potential for herb-drug interactions (HDIs) in pregnant women and to explore possible herb-drug interactions and their potential clinical significance.

**Methods:**

A cross-sectional survey of women during early pregnancy or immediately postpartum in North-East Scotland. Outcome measures included; Prescription medicines use excluding vitamins and potential HDIs assessed using Natural Medicines Comprehensive Database.

**Results:**

The survey was completed by 889 respondents (73% response rate). 45.3% (403) reported the use of at least one prescription medicine, excluding vitamins. Of those taking prescription medicines, 44.9% (181) also reported concurrent use of at least one HNP (Range 1–12). A total of 91 different prescription medicines were reported by respondents using HNPs. Of those taking prescription medicines, 44.9% (181) also reported concurrent use of at least one HNP (Range 1–12). Thirty-four herb-drug interactions were identified in 23 (12.7%) women with the potential to increase the risk of postpartum haemorrhage, alter maternal haemodynamics, and enhance maternal/fetal CNS depression.

Almost all were rated as moderate (93.9%), one as a potentially major (ginger and nifedipine) and only one minor (ondansetron and chamomile).

**Conclusion:**

Almost half of pregnant women in this study were prescribed medicines excluding vitamins and minerals and almost half of these used HNPs. Potential moderate to severe HDIs were identified in an eighth of the study cohort. Healthcare professionals should be aware that the concurrent use of HNPs and prescription medicines during pregnancy is common and carries potential risks.

## Background

Complementary and alternative medicine (CAM) use is reportedly increasing worldwide, with data from the United Kingdom (UK), Europe and United States (USA) confirming that herbal and natural products (HNP) are the most frequently used form of CAM therapy [1–5]. Although HNP have been used widely for thousands of years there is a lack of robust data to support their medical use. Despite the lack of safety and efficacy data the public do not appear to be deterred from using them with 5.9%–48.3% of Europeans and 12–18% of North Americans reportedly using HNP [4, 5]. Herbal medicines are often promoted as harmless and users typically cite that HNPs provide a safe alternatives to pharmaceutical medicines, as a reason for use [6]. However the use of HNP has been associated with adverse effects due to direct chemical toxicity, herb-drug and herb-herb interactions, incorrect dosing, toxic constituents (renal or hepatic toxins), and product adulteration or contamination with toxic metals, microbes, or pollutants [7–18].

Women are recognised to be the major users of HNP for treatment of disease and maintenance of health [3–5, 19, 20], and this widespread use also extends into pregnancy, where reportedly between 10 and 74% of pregnant women in Australia, Europe, UK and the USA use these products [21–30]. Recent data suggest that approximately 40% of UK pregnant women use HNP to treat pregnancy related problems such as nausea and vomiting or as nutritional supplements to aid fetal development [26, 27].

Although the potential risks associated with the use of prescription medicines during pregnancy are well recognised, data from Australia, Europe and North and South America, suggest that 12–81% of pregnant women are prescribed medicines by their healthcare professional who may be unaware of possible HNP use by their patient [31–35]. Therefore, during pregnancy many women may be using both HNP and prescribed medicines resulting in a risk of an herb-drug interaction which may be significant [36, 37]. The current level of concomitant HNP and prescription medicine use in the UK is unclear, studies from North America, Europe, Asia and Africa suggest that between 2.5%–13% of pregnant women use herbal and natural products together with prescribed medicines [38–41]. Currently, there are limited published data from the UK reporting the levels of HNP use together with prescription medicines during pregnancy and the possible resultant herb-drug interactions.

The aims of this study were to assess the prevalence and characteristics of HNP use in pregnant women also taking prescription medicines and to explore the possible herb-drug interactions and their potential clinical significance.

### What is known about this subject

- Women are recognised to be the major users of HNP, for treatment of disease and maintenance of health, and in high, middle and low income countries this widespread use also extends into pregnancy.
- Given that 12–81% of pregnant women use prescription medicines, a significant number are likely to also be using HNP with the risk of potential herb-drug interactions.

### What this study adds

- Almost half of all pregnant women in our study population are prescribed medicines and almost half of these use HNPs.
- The potential for moderate to severe herb-drug interactions were identified in an eighth of women.

## Methods

Data assessing the use of CAM collected from women attending for their mid-trimester (18–21 weeks) scan (*n* = 332) and women, within the first 24 h following a live birth, admitted to the postnatal unit (*n* = 557) at the Royal Aberdeen Maternity Hospital, North-East Scotland were combined. Data collection was completed in 2012 and study methods have been reported in detail elsewhere [26, 27]; brief study details are given for completeness. Questionnaires based on the findings of a systematic review assessing the quality of relevant study methodologies [6] were given to women attending the antenatal clinic for their mid trimester scan or on the postnatal ward *following delivery*. The questionnaire was written in English only and tested for face and content validity by a panel of healthcare professionals, pregnant and postpartum women. The final questionnaire contained four sections comprising: health and medication use during pregnancy (4 items); personal use of CAM therapies (7 items, extensive checklist of CAM modalities and products); attitudes toward CAM use during pregnancy (6 items); and demographics (5 items). Questions were a mix of closed and Likert statements. As the questionnaire was anonymous no reminders were issued. To optimise the return rate the questionnaire did not seek information on the quantity, timing, dose or duration of herb and food supplements taken. From the combined study populations all women reporting the use of prescribed medicines and/or herbal or natural products were identified. To help participants to identify correctly the products or modalities considered as herbal or natural products a check list was provided listing 40 herbal and natural products from the Medicines and Healthcare-Products Regulatory Agency with space for other products which the respondent might be using. Prescribed medicines were recorded by asking the respondent “Have you taken any prescribed medicines during this pregnancy? If yes, please write the names of all the medicines you have been prescribed.”

### Statistical analysis

Data were coded and entered into an SPSS 22.0 database. Descriptive statistics were used to analyse the data and provide respondent profile. Chi-square was used to test the associations between age, education and the use of herbal or natural products; *p* < 0.05 was considered statistically significant.

### Herb-drug interaction analysis

The potential for herbal and natural product interaction with prescribed medicines was assessed using the Natural Medicines Comprehensive Database [42]. This database reports and grades known interactions as major (do not use combination; contraindicated; strongly discourage patients from using this combination; a serious adverse outcome could occur), moderate (use cautiously or avoid combination; warn patients that a significant interaction or adverse outcome could occur), and minor (be aware that there is a chance of an interaction; advise patients to watch for warning signs of a potential interaction) together with a description of the mechanism underlying the interaction.

### Ethics statement

This research was approved by National Health Service North of Scotland Research Ethics Committee and National Health Service Grampian Research and Development Committee on June 27, 2011 (REC 11/AL/0094). As the Ethics Committee required the survey questionnaires to be fully anonymous and returned directly by respondents in post-paid envelopes with no record of identifiable data, written consent to participate was deemed unnecessary.

## Results

A total of 889 respondents completed the questionnaire on herbal and natural products use (332 antenatal, 557 immediate postnatal, response rates 66% and 80% respectively, study period March–August 2012). Of the 889 respondents, 45.3% (403) reported the use of at least one prescription medicine, excluding vitamins and minerals. Of the 403 pregnant women reporting the use of prescription medicines, 44.9% (181) also reported the concurrent use of at least one herbal or natural product (Range 1–12), equating to 20.4% of the total study population.

The demographics of women reporting concurrent use of HNP and prescription medicines (“Users”) and those reporting prescription medicines only (“Non-Users”) are reported in Table 1. “Users” were significantly older (*p* < 0.05) and had a higher education status (*p* < 0.05) than “non-users”. “Users” in the early pregnancy group reported the use of 16 different herbal and natural products, of which ginger was the most commonly cited (35.6%), followed by chamomile (23.2%), cranberry (20.5%), and fish oil (12,3%). In the late pregnancy group, a total of 20 herbal and natural products were reported, of which raspberry was most frequently used (42.5%) followed by cranberry (26.7%), ginger (23.7%), fish oil (14.8%), and chamomile (11.8%). The majority of “users” (57.1%) reported the use of one HNP, 20.1% two, 15.8% three and 7.0% four or more HNPs (Range 1–6).

**Table 1:** Demographics of women reporting concurrent use of prescription medicines with herbal and natural products (Users) and women reporting prescription medicine use only (Non-Users) (*n* = 403)

	“Users” (*n* = 174, *n* (%))	“Non-Users” (*n* = 229, *n* (%))	*p*-value (Chi-square)
Age (years)			
15–24	20 (11.5)	35 (15.3)	
25–34	101 (58)	149 (65)	
≥ 35	53 (30.4)	45 (19.7)	<0.05
Education level			
University	99 (56.9)	108 (47.2)	
College	52 (29.8)	63 (27.5)	
Secondary school	23 (13.2)	56 (24.4)	<0.05
Not stated		2 (0.8)	
Number of prescribed medicines			
Median (IQR)	1 (1–2)	1 (1–2)	
Range	1–12	1–10	
Number of herbal/natural products			
Median (IQR)	1 (1–2)		
Range	1–6		

### Prescription medicine use by “users”

The use of a single prescription medicine was reported by over half (54%) of “users”, two medicines by 26%, three by 10%, four by 5% and five or more by 5% (range 1–12). A total of 91 different prescription medicines were reported by respondents, the top drug groups in order of frequency were: antibacterials (18.3%); opiate analgesics (8.3%); anti-emetics (7.1%); thyroid replacement (7.1%); non-steroidal anti-inflammatory drugs (NSAIDs) and aspirin (6.6%); laxatives (6.6%); antacids (5.8%), beta-2-agonists (5%); non-opiate analgesics (paracetamol) 4.6%, H2 antagonists (4.1%); and antidepressants (4.1%).

The majority of respondents (56.9%) reported the use of one HNP with prescribed medicines, 20% reported two, 16% three, 5% four and 2% five or more (range 1–6).

### Potential herbal and natural products-prescription medicine interactions

Eight distinct HNP entities were identified as having the potential to cause interactions with concurrent prescription medicines. These were aloe, chamomile, cranberry, fish oil, ginger, ginseng, grapefruit, and sage. A total of 34 potential herb-drug interactions were identified in 23 (12.7%) of the “users”. Of the 34 potential interactions, almost all were rated as moderate (93.9%), one as a potentially major (ginger and nifedipine) and only one minor (ondansetron and chamomile). Two underlying mechanisms for the potential interactions were pharmacodynamic, accounting for 54.5% and pharmacokinetic via inhibition of cytochrome P450 enzymes or decreased drug absorption, the remainder.

The prescribed drug classes with the potential for interaction with HNP were antithrombotic agents (7), antihypertensive agents (6), antidiabetic agents (5), hypnotics and anxiolytics (4), opiate analgesics (4), NSAIDs (3), hormonal therapies (2, prevention of miscarriage), proton pump inhibitors (1), insulin (1) and anti-emetics (1). The HNP and interacting prescription medicines together with the potential severity and mechanism of the interaction are reported in Table 2.

**Table 2:** Potential herb-drug interactions (HDIs), severity and mechanism identified among respondents reporting the use of herbal and natural products with prescription medicines (*n* = 181)

Herbal/Natural Product	Prescribed Medicine	Interaction Rating	Mechanism of Interaction
Aloe (12)	Insulin (1)	Moderate	Additive effects, lower blood glucose levels lead to increased risk of hypoglycaemia.
Chamomile (29)	Diazepam (1)	Moderate	Additive effects of benzodiazepines, lead to increased risk of CNS depression, psychomotor impairment.
	Propranolol (1)	Moderate	Inhibition of CYP1A2 and CYP2D6 (in vitro and in vivo). Increase levels of drugs metabolized by this enzyme.
	Diclofenac (1)	Moderate	Inhibition of CYP2C9 (in vitro). Increase levels of drugs metabolized by this enzyme.
	Ondansetron (1)	Minor	Inhibition of CYP1A2 (in vitro and in vivo). Increase levels of drugs metabolized by this enzyme.
	Chlorpromazine (1)	Moderate	Inhibition of CYP2D6 (in vitro). Increase levels of drugs metabolized by this enzyme.
	Dyhidrocodeine (2)	Moderate	Additive effects of codeine, lead to increased risk of CNS depression, psychomotor impairment.
	Co-codamol (1)	Moderate	Additive effects of codeine, lead to increased risk of CNS depression, psychomotor impairment.
Cranberry (42)	Diazepam (1)	Moderate	Inhibition of CYP2C9 (in vitro), contradictory results in clinical research. Increase levels of drugs metabolized by this enzyme.
	Diclofenac (1)	Moderate	Inhibition of CYP2C9 (in vitro), contradictory results in clinical research. Increase levels of drugs metabolized by this enzyme
Fish oil (24)	Progesterone pessaries (2)	Moderate	Progesterone may interfere with the triglyceride lowering effects of fish oils.
	Dalteparin (2)	Moderate	Additive effects, lead to increased risk of bleeding.
	Enoxaparin (1)	Moderate	Additive effects, lead to increased risk of bleeding.
	Labetolol (2)	Moderate	Additive effects with antihypertensive, lead to increased risk of hypotension.
	Methyldopa (1)	Moderate	Additive effects with antihypertensive, lead to increases risk of hypotension.
Ginger (50)	Metformin (1)	Moderate	Additive effects, might increase insulin level and/or decrease blood glucose levels, may lead to hypoglycemia.
	Insulin (2)	Moderate	Additive effects, might increase insulin level and/or decrease blood glucose levels, may lead to hypoglycemia.
	Aspirin (4)	Moderate	Inhibit thromboxane synthetase and decrease platelet aggregation lead to increased risk of bleeding.
	Nifedipine (1)	Major	Significantly inhibits platelet aggregation, synergetic effects, may lead to potential cardiovascular and cerebrovascular complications.
Ginseng (4)	Metformin (1)	Moderate	Additive effects, might increase insulin level and/or decrease blood glucose levels, may lead to hypoglycemia.
	Diazepam (1)	Moderate	Additive effects of benzodiazepines, lead to increased risk of CNS depression, psychomotor impairment
Grapefruit (8)	Itraconazole (1)	Moderate	Might affect absorption and might increase or decrease itraconazole levels.
	Propranolol (1)	Moderate	Inhibition of CYP1A2 (preliminary evidence). Increase levels of drugs metabolized by this enzyme.
	Diclofenac (1)	Moderate	Inhibition of CYP2C9 (preliminary evidence), contradictory results in clinical research. Increase levels of drugs metabolized by this enzyme.
	Omeprazole (1)	Moderate	Inhibition of CYP2C19 (preliminary evidence), contradictory results in clinical research. Increase levels of drugs metabolized by this enzyme.
Sage (1)	Co-codamol (1)	Moderate	Inhibition of CYP2D6 (in vitro). Increase levels of drugs metabolized by this enzyme.

## Discussion

Almost half of our study population were taking prescription medicines and just under half of these reported the concurrent use of at least one herbal or natural product. We identified potential herb-drug interactions in an eighth of women who concurrently used both HNP and prescription medicines, with one interaction being reported as potentially severe, one as mild and the remainder as moderate in severity.

Herbal medicines are the most common form of CAM used worldwide [43] with women reportedly the main users [20]. Despite the lack of robust safety and efficacy data for HNPs, recent studies suggest that approximately 40% of pregnant women in the UK report herbal and natural product use during early and or late pregnancy [26, 27]. In this study we identified that almost half of pregnant women were using prescription medicines, which is in agreement with the findings of previous studies reporting that 12–81% of pregnant women use prescription medicines [31–35].

Although the current level of concomitant HNP and prescription medicine use in the UK is unclear, studies from North America, Sweden, Kenya and Iran suggest that between 2.5%–13% of pregnant women use HNP together with prescribed medicines [38–42]. The levels of HNP and prescribed medicines use reported in the published literature are highly variable and generally lower than those observed in our study; however, there are significant methodological differences in the manner in which data was collected from these different study populations, which make direct comparison difficult [44].

In this study we identified 34 potential HNP-drug interactions, predominantly classified as moderate with one mild and one severe, affecting 12.7% of HNP users. This would suggest that approximately 1 in 20 of all pregnant women in the UK, who use HNP, are at risk from a potential herb-drug interaction. While the potential for herb-drug interactions has been assessed in patients with a variety of disease states, only one study has reported on the prevalence and severity of such interactions in the pregnant population [38]. Moussally et al. identified that while only 9% of pregnant Canadian women used HNPs, 69% of these consumed at least one prescribed medicine [38]. Their study noted potential herb-drug interactions for more than a third of drug-herb combinations of which more than 1:4 had the potential to lead to serious adverse effects. In our study we identified that approximately 45% of women who reported herbal and natural product use also used prescription medicines, however we observed only one potentially serious interaction. The reasons for the lower prevalence of interactions, especially severe interactions, in our study population is not clear, however it is likely due to differences in the herbal and natural products used by the two study populations.

In our study four herbal and natural products, ginger, chamomile, grapefruit and fish oil, which are frequently used for the treatment of pregnancy related issues, accounted for 82% of all potential interactions. Moussally et al., however, reported that green tea, which did not appear in the top 10 herbs used by our cohort, accounted for 30% of all herb-drug interactions in a Canadian population [38]. Although we have identified potential rather than actual interactions, these interactions may give rise to clinically significant adverse events, especially during labour and birth. Such herb-drug interactions have been previously reported to give rise to increased risk of bleeding, alteration of maternal haemodynamics and increased risk of central nervous system depression both maternal and fetal/neonatal [45–47]. Herbal supplements such as valerian, kava and chamomile when administered with opiate analgesics, which were the second largest prescribed drug group in our study cohort, may lead to increased central nervous system depression. Similarly, NSAIDs, particularly aspirin, have the potential to interact with herbal supplements which also possess antiplatelet activity, such as ginkgo, garlic, ginger, and ginseng, or with herbs containing coumarin such as chamomile, fenugreek and red clover, to enhance the risk of bleeding. We did not look specifically at the use of over the counter medicines (OTC), however in this study 11 women were prescribed paracetamol, a medicine which is commonly bought over the counter in pharmacies or supermarkets. The true use of paracetamol in our study population is therefore likely to have been significantly greater than reported. While many of the public regard paracetamol as a mild analgesic it does have the potential to interact with a number of herbs and food supplements such as Echinacea to increase the risk of bleeding, hepatotoxicity and nephrotoxicity.

### Strengths and limitations

Respondents were provided with detailed checklists for specific herbal and natural products to ensure correct reporting of use. We have demonstrated previously that using such a checklist, rather than an open question about herbal or natural product use, produces a nine-fold increase in the number of respondents who report herbal or food supplement use [44].

A limitation of our study however is the use of self-reported data and sampling from one centre, which may possibly limit generalizability, however the demographic makeup of our respondents is identical to that of the total pregnant population of England and Wales [48] indicating that our results are likely to be generalisable to the UK. To ensure an optimal response rate the questionnaire did not ask about the quantity, timing, dose or duration of herb and food supplements therefore it is possible that women stopped taking their herbal medicines when prescribed an interacting medicine. Furthermore we did not collect data on medicines purchased over the counter (e.g. paracetamol), which may have led to an underestimation of the number of potential herb-drug interactions. Although we used a comprehensive international information source to identify potential interactions, it is possible that further interactions may have been identified had other information sources been used or that further herb-herb or herb-drug interaction are as yet unrecognised or unreported.

## Conclusion

In the UK, HNPs are used by almost half of pregnant women, although most are unaware that they are using herbal medicines. While HNPs are often promoted as harmless and users may believe that HNPs provide a safe alternatives to pharmaceutical medicines, many HNPs contain active ingredients, which have the potential to interact with prescribed medication either directly or via modification of metabolism. In our study almost half of pregnant women were using prescribed medicines, excluding vitamins and minerals, and almost half of these also used HNPs. Because of the large number of HNPs available to the public it is not surprising that the toxicology for many is limited, however despite this we identified potential herb-drug interactions of moderate severity in approximately one in eight pregnant women using HNPs. While we were able to identify potential HDIs we were unable to determine whether individuals were affected clinically, however as there are approximately 700,000 births in the UK annually and approximately one fifth of these women use prescription and herbal medicines concurrently the number affected by HDIs is not insignificant. Healthcare professionals and those recommending HNPs should be aware that the concurrent use of HNPs and prescription medicines during pregnancy is common and may place the mother and fetus at potential risk.

## Abbreviations

CAM: Complementary and Alterative Medicine
CNS: Central Nervous System
HDI: Herbal Drug Interaction
HNP: Herbal and Natural Product
NSAID: Non-Steroid Anti-Inflammatory Drug
UK: United Kingdom
USA: United States
